# Network of proteins, enzymes and genes linked to biomass degradation shared by *Trichoderma* species

**DOI:** 10.1038/s41598-018-19671-w

**Published:** 2018-01-22

**Authors:** Maria Augusta Crivelente Horta, Jaire Alves Ferreira Filho, Natália Faraj Murad, Eidy de Oliveira Santos, Clelton Aparecido dos Santos, Juliano Sales Mendes, Marcelo Mendes Brandão, Sindelia Freitas Azzoni, Anete Pereira de Souza

**Affiliations:** 1 Center for Molecular Biology and Genetic Engineering (CBMEG), University of Campinas (UNICAMP), Campinas, SP Brazil; 2 University Unit of Biology, West Zone State University (UEZO), Rio de Janeiro, RJ Brazil; 3 Bioethanol Science and Technology Laboratory (CTBE), Brazilian Center of Research in Energy and Materials (CNPEM), Campinas, SP Brazil; 4 Department of Plant Biology, Biology Institute, University of Campinas (UNICAMP), Campinas, SP Brazil

## Abstract

Understanding relationships between genes responsible for enzymatic hydrolysis of cellulose and synergistic reactions is fundamental for improving biomass biodegradation technologies. To reveal synergistic reactions, the transcriptome, exoproteome, and enzymatic activities of extracts from *Trichoderma harzianum, Trichoderma reesei* and *Trichoderma atroviride* under biodegradation conditions were examined. This work revealed co-regulatory networks across carbohydrate-active enzyme (CAZy) genes and secreted proteins in extracts. A set of 80 proteins and respective genes that might correspond to a common system for biodegradation from the studied species were evaluated to elucidate new co-regulated genes. Differences such as one unique base pair between fungal genomes might influence enzyme-substrate binding sites and alter fungal gene expression responses, explaining the enzymatic activities specific to each species observed in the corresponding extracts. These differences are also responsible for the different architectures observed in the co-expression networks.

## Introduction

Enzymatic hydrolysis is currently used as a decisive step towards the biotechnological use of biomass. The biological degradation of the carbohydrates within the biomass is achieved using multiple enzymes in defined ratios to convert the carbohydrates to their monomer sugars. This is followed by the fermentation of these sugars into bioethanol. The enzymes cooperate in a synergistic fashion to degrade the substrate^1^. With the objective of understanding and reproducing the efficiency of these reactions, the scientific community has performed numerous studies prospecting and characterizing the enzymes required to degrade various components of lignocellulose, the impact of pretreatments on the lignocellulose components and the enzymes required for degradation. Many factors affect the enzymes and the optimization of the hydrolysis process, such as enzyme ratios, substrate loadings, enzyme loadings, inhibitors, adsorption, surfactants, degrees of synergy and yield. Through enzymatic reactions, fungi are able to degrade the long polymer chains that constitute the main components of biomass, including cellulose, hemicellulose and lignin, leading to sugars that can be fermented to ethanol and other products^2–7^. The focus of this work was to investigate the kinds of synergistic reactions promoted by the fungi responsible for biomass structure challenges.

Species in the filamentous ascomycete genus *Trichoderma* are among the most commonly isolated saprotrophic fungi. They are frequently found in soils and growing on wood, bark, other fungi and innumerable other substrates^8^. *Trichoderma reesei* is the most widely employed cellulolytic organism in the world. The generation of high cellulase-producing mutants from the wild-type *T. reesei* strain QM6 have produced the hypercellulolytic strain RUT-C30^9^, although high levels of cellulase are also produced in other species from this genus^10,11^. The use of *Trichoderma harzianum* species in biotechnology has been explored by examining the biocontrol capacity of this species^12,13^. Most research on mycoparasitism has been performed in only a few of these species, including *Trichoderma harzianum*, *Trichoderma atroviride, Trichoderma virens*, *Trichoderma asperellum* and *Trichoderma asperelloides*^14,15^. These species promote the death of prey through the synergistic actions between secondary antifungal metabolites and cell wall-hydrolytic enzymes that are secreted by *Trichoderma* spp. The genetic mechanisms underlying the perception, control and production of important enzymes have been investigated, and the functions of some of the genes involved have been determined^16^. More recently, obtained data have allowed exploration of the diversity of cellulases and accessory enzymes produced by these species, which hydrolyse different types of carbohydrates^17–20^. The efficiency of hydrolytic enzymes appears to be linked to the degree of synergism between them and the precise design of catalytic regions that may affect the reaction rate with the substrate^21^. Studies of enzymes produced from *Trichoderma* are necessary to find more efficient and low-cost enzymes, which are useful in different steps of the hydrolytic process of biomass depolymerization. The efficiency of hydrolytic enzymes mainly seemed to be linked to the degree of synergism between them and to the precise design of catalytic regions, which may affect the rate of reaction with the substrate. Thus, the study of different fungal species must provide valuable information on protein and gene structures, clarifying the regulatory mechanisms related to hydrolysis, and the principal proteins shared by different species that favour the enzymatic reactions. In this context, this study explored genetic mechanisms underlying the expression and secretion of hydrolytic enzymes used by *Trichoderma* spp. to degrade cellulose by considering a common ancestor and differentiation processes that originated in the various studied species.

## Results

### Phylogenetic relationships and enzymatic activities profiles

**Figure 1:**
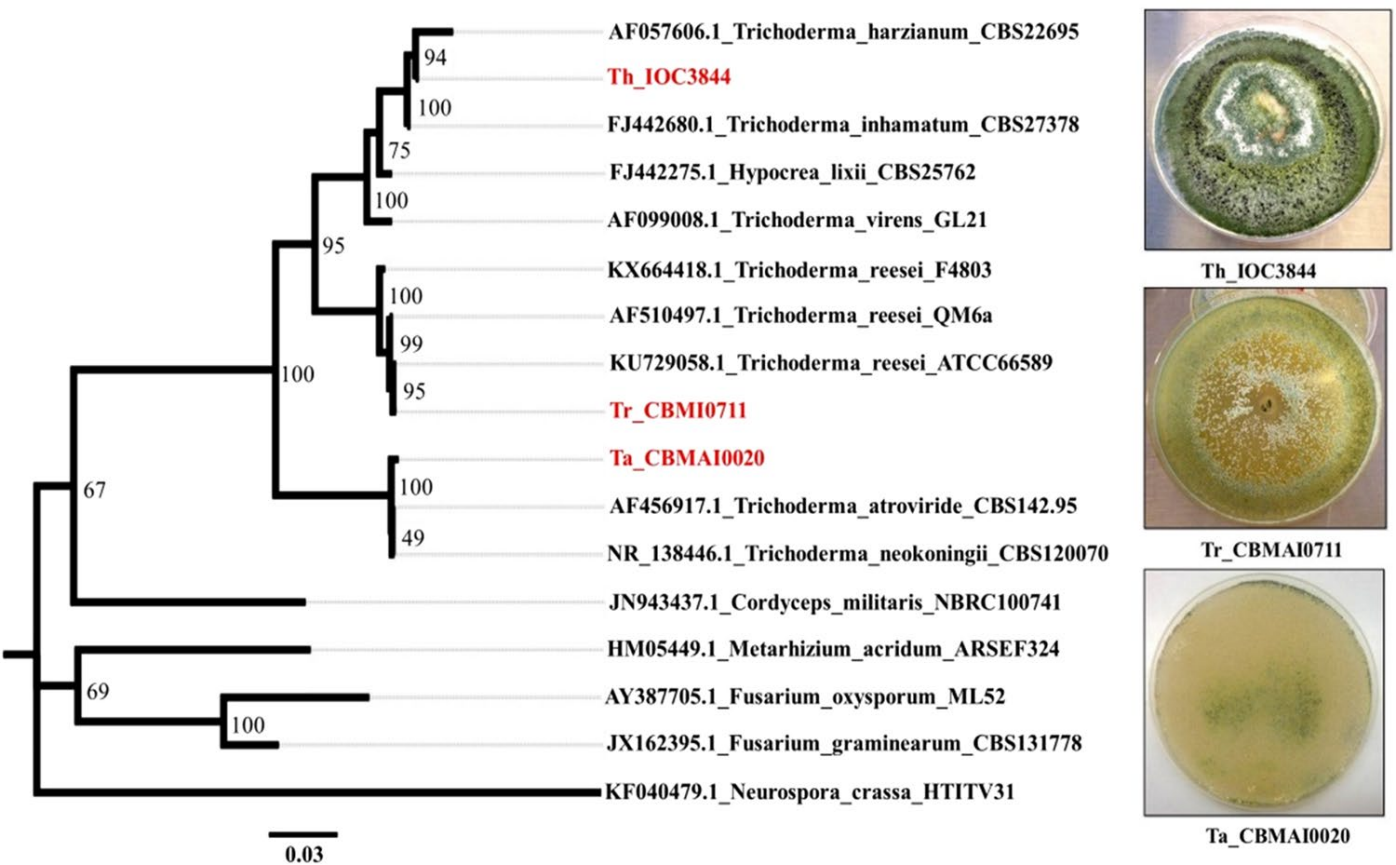
Phylogenetic tree of *Trichoderma* spp. (Th, Ta and Tr). The internal transcribed spacer (ITS) region was used for identity confirmation and phylogenetic relationships of the studied *Trichoderma* species with taxonomically related fungi.

**Figure 2:**
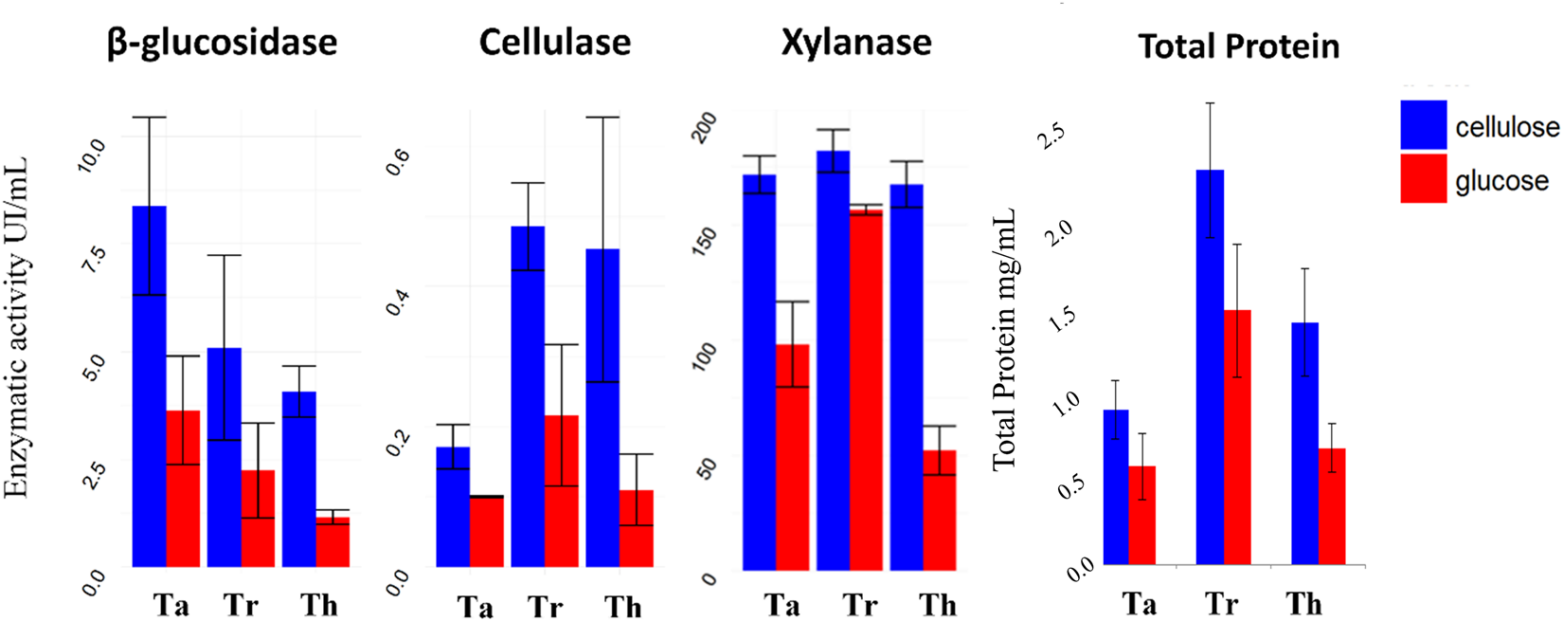
Cellulase, xylanase and β-glucosidase enzymatic activities and protein contents in culture supernatants. *Trichoderma* cultures were induced by glucose or cellulose treatment, and enzymatic activities and protein contents were measured after 96 h of growth.

The genus *Trichoderma* is clearly separated from other types of degrading fungi. The phylogenetic tree shows a high phylogenetic proximity between *T. harzianum* (Th) and *T. reesei* (Tr) (with bootstrap support of 95%), and these species presented a close relationship with other strains in the same genus (Fig. 1). As shown in the tree presented in Fig. 1, the *T. atroviride* (Ta) strain CBMAI0020 shared the closest affinity with Ta CBS142.95, with 100% bootstrap support. Although the similarity between species shows that they share a largely common genetic background, small genetic differences can be detected when comparing their enzymatic performances. The phylogenetic analysis shows a differentiation process for Ta, while Th and Tr exhibited the closest phylogenetic relationships with Th CBS226-95 and Tr ATCC66589, respectively. The enzymatic activity of aqueous extract was measured to demonstrate the individual potential for production of hydrolytic enzymes. We determined filter paper activity (FPA) related to cellulase enzyme activities, xylanase and β-glucosidase enzyme activities in the culture supernatants after 96 h of fermentation. This was the same point for RNA extraction and extracellular protein detection, leading to distinct enzymatic activity profiles (Fig. 2). The carbon source plays an important role in the production of enzymes. In the present study, we used cellulose and glucose during the growth, promoting the expression of different sets of genes as the fungus seeks to adapt to different environments. The enzymatic activity reaches high levels at 96 h and was expected to find the complete set of transcripts relative to the active gene system for degradation at that time. The cellulase activity observed in the samples grown on glucose was significantly lower for all strains in comparison with growth on cellulose. The Ta strain displayed lower enzymatic activity than the other strains, all of which were considered to exhibit statistically equal cellulase activities during growth on cellulose. The experiment was conducted with biological triplicates and showed a sharp standard deviation, thus, we could not identify real differences between the Tr and Th strains. However, the different carbon sources, glucose and cellulose, generated different enzymatic responses in each strain. The xylanase and β-glucosidase activities suggest enzymatic activities towards hemicellulose compounds. Even under fermentation conditions in the absence of pure pentoses, differences in enzymatic activities were observed in the presence of cellulose. The xylanase activity was higher in all strains grown on cellulose, the activity was statistically equal in the Ta, Th and Tr strains. The β -glucosidase activity was increased by growth on cellulose in the Th and Ta strains but showed no significant difference in the Tr cultures (Fig. 2). The total amount of secreted proteins appeared to be greater in the cellulose cultures for all species. The Th and Tr strains presented the highest levels of total protein in the extracellular extracts (Fig. 2).

### Qualitative and quantitative correlation of the exoproteome via mass spectrometry

**Figure 3:**
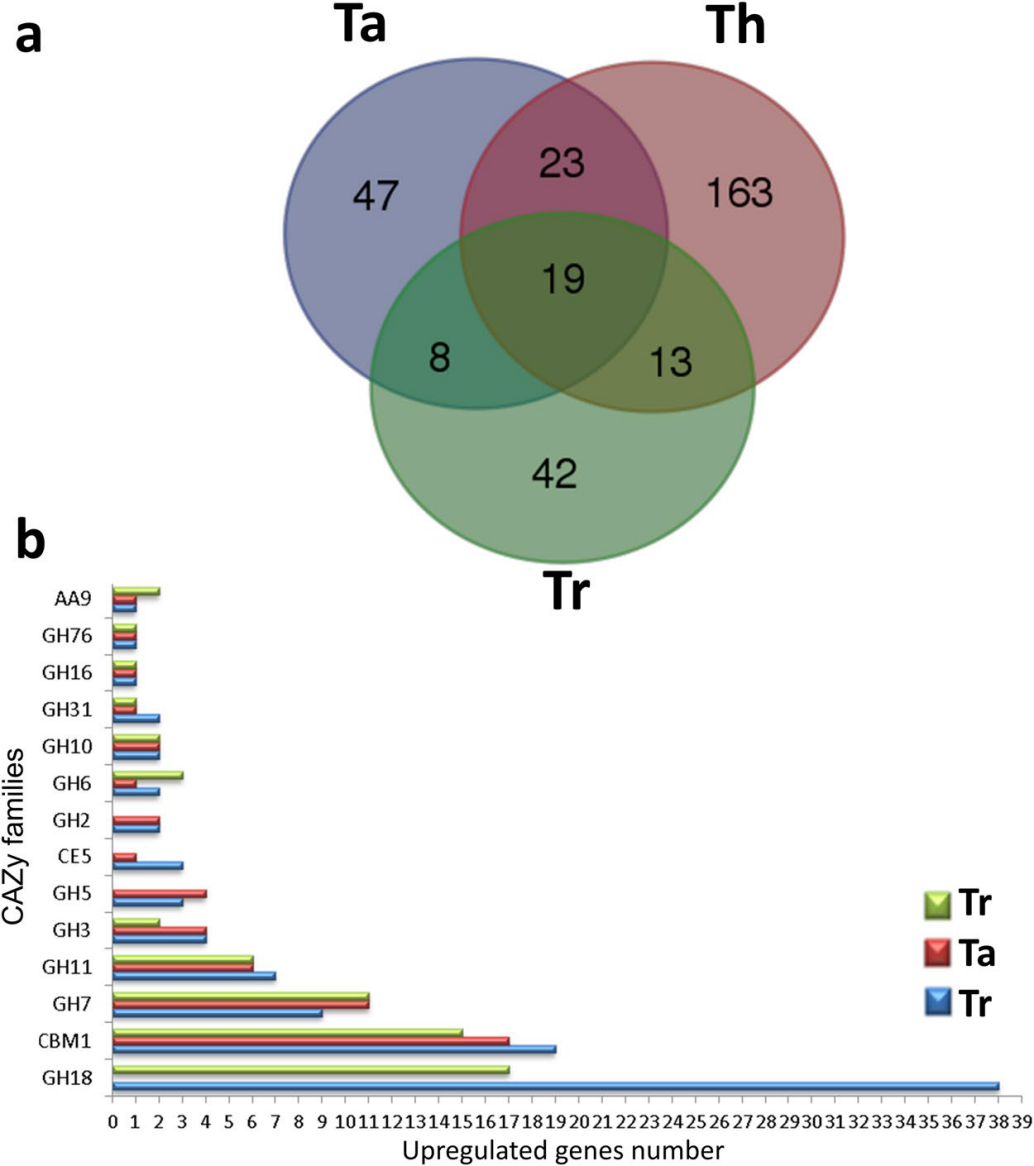
Protein classification by exoproteome analysis. (**a**) Venn diagram comparing proteins detected in the cultures of *Trichoderma* species. (**b**) Distribution of CAZymes among the secreted proteins of Tr, Ta, and Tr under both fermentative conditions.

Through mass spectrometry analysis, it was possible to determine the proteins present in the extract, which had been secreted and were free in the culture medium. These proteins have fundamental importance because they could be considered active at the exact moment of fermentation and responsible for the enzymatic activities detected in the extract and described in this report. A total of 397 proteins were identified using the *Trichoderma* protein data available in the UniProtKB AC/ID database, including 82 proteins from Tr cultures, 97 proteins from Ta cultures and 218 proteins from Th cultures. Figure 3 and Supplementary Information 1 and 2 describe the detected proteins in detail, the ratios of detected proteins between the treatments, and correlations between the presence/absence of proteins in different species. The identified protein profiles were compared with the aim of detecting similarity between the samples of each species, and the proteins were further classified as CAZymes (Fig. 3). We identified the set of proteins specific to or shared by species in the exoproteome (Supplementary Information 2). Nineteen of the proteins were identified in all species, and these were related to different biological mechanisms of biomass depolymerisation. GHs were identified according to the literature, with the majority of these proteins belonging to the GH18 famil^22^. Among these families, which primarily included chitinases (EC 3.2.1.14) and endo-β-N-acetylglucosaminidases (EC 3.2.1.96), 38 Th proteins and 17 Tr proteins were identified, and notably, none of these proteins were detected in the Ta strain. Other observed families included CE or carbohydrate esterases and GT or glycosyltransferases. A bimodular GH7 protein, carbohydrate-binding module family 1 (CBM1), was detected in all species cultured in cellulose and only in the Tr strain cultured with glucose.Ta exhibited a different enzymatic profile; therefore, proteins detected only in Ta may respond by increasing β-glucosidase activity (Supplementary Information 2). Forty-two proteins were identified, including β-glucosidases. Some proteins appear to be very similar to β-glucosidase (EC 3.2.1.21), including A0A060DHV5_9HYPO, which shares 94.6% identity with G9NS06_HYPAI and 91.8% identity with G9 MUM1_HYPVG. For example, GUX1_HYPJE, an exoglucanase 1 (EC 3.2.1.91) from the GH7 family, was found to be secreted by all species. Very similar proteins are contained in databanks, some of which differ by only one base pair but still receive different identification codes. Such similarity was observed in the present study, and only structural and functional tests can clearly determine the exact identity of a protein. However, these differences must be considered.

### RNA-Seq analysis and exoproteome integration

**Figure 4:**
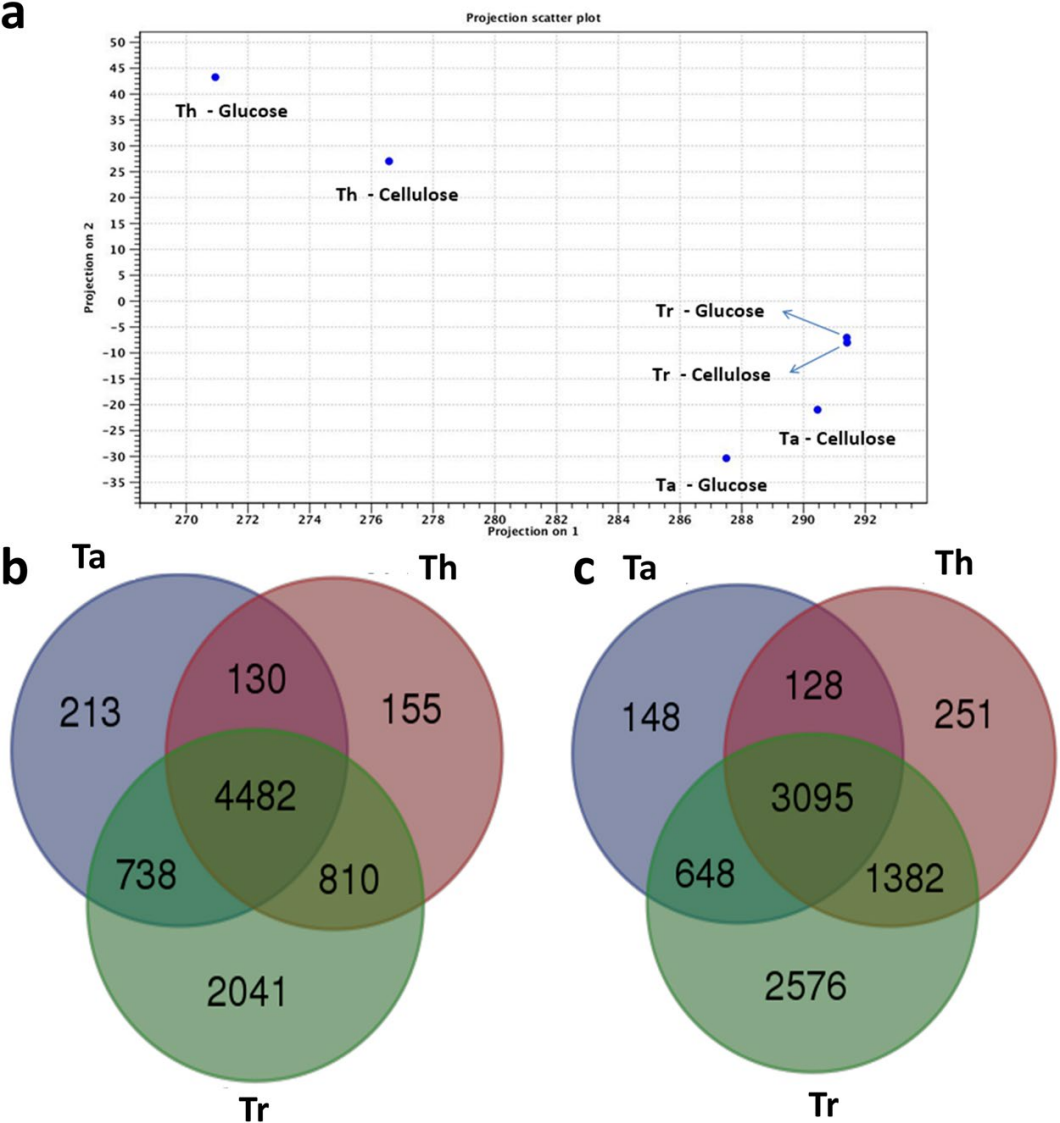
Transcriptome content of each species according to reference genome *T. harzianum* T6776. (**a**) Principal component analysis (PCA) of transcriptome mapping according to species and growth conditions. (**b**) Venn diagram comparing identified genes with expression higher than zero under cellulose growth. (**c**) Venn diagram comparing identified genes with expression higher than zero under glucose growth.

**Figure 5:**
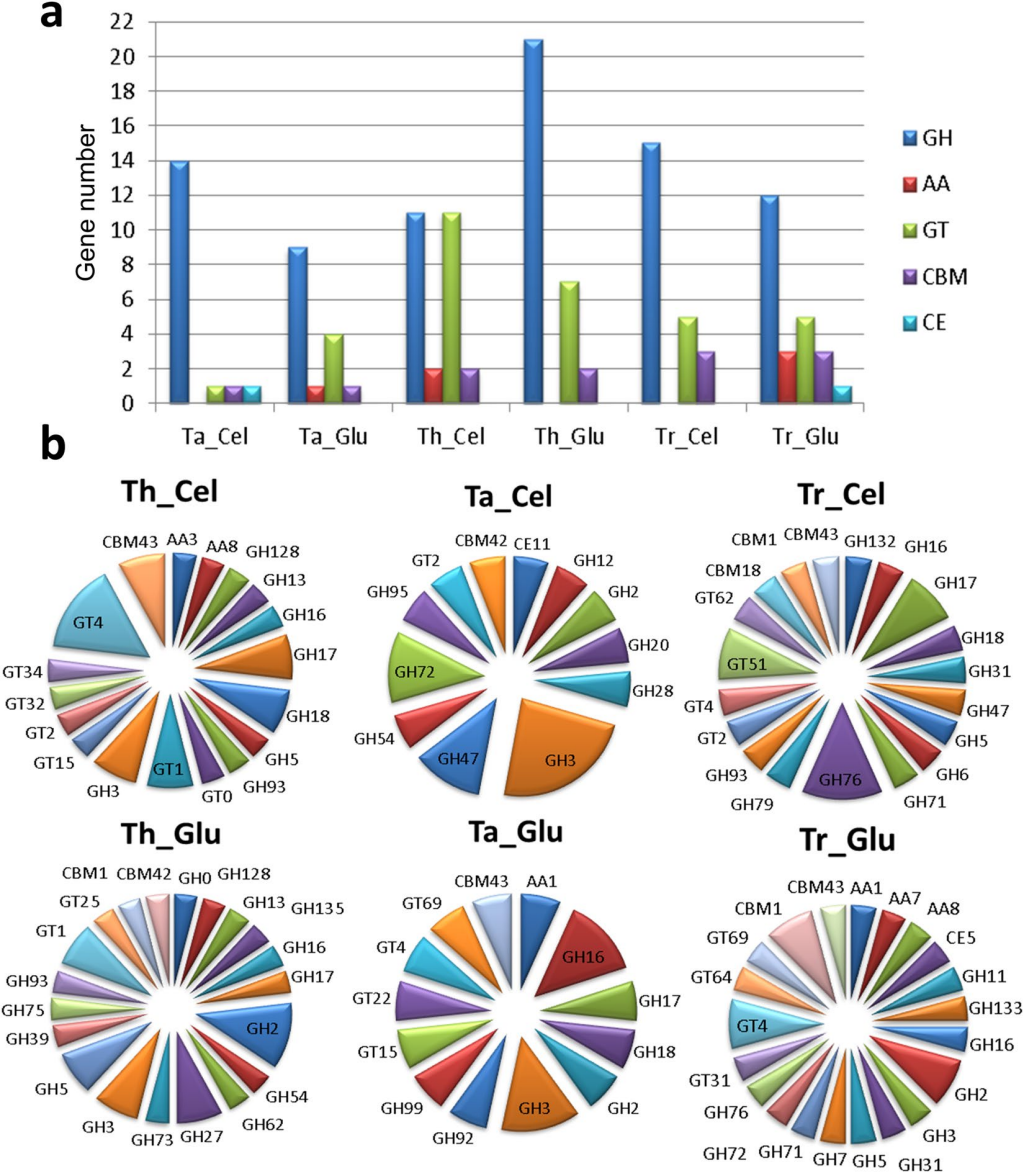
CAZy classification of transcriptome upregulated genes. (**a**) Upregulated genes from the principal CAZy families (CAZy families: GH – glycoside hydrolase, AA - auxiliary activities, GT – glycoside transferase, CBM - carbohydrate-binding module, CE - carbohydrate esterase) according to species (Th, Ta and Tr) and treatment (cellulose and glucose) (**b**) Distribution of CAZy families among the upregulated genes identified in Th, Ta and Tr under growth on cellulose (Cel) and glucose (Glu).

The complete lists of the mapped genes, mapping statistics and RNA-Seq profiles of each species are provided in Supplementary Information 6. After mapping the reads from each species against the *T. harzianum* T6776 reference genome, a principal component analysis (PCA) was performed to determine the similarities and differences in gene expression between the species and treatments. The PCA results showed three distinct groups across the transcriptome profiles, with a higher similarity between treatments than between species. Only the Tr strain showed no major differences between treatments. According to the PCA, the Tr and Ta strains displayed higher levels of transcriptome similarity (Fig. 4a). Venn diagrams were constructed based on the similarities between the classified genes from all species. The genes that exhibited expression levels greater than zero were identified and compared using the corresponding intersection sets for the cellulose and glucose treatments in the different species (Fig. 4b and c). A greater number of genes was identified under cellulose growth conditions (8569 genes) than under glucose growth conditions (8228 genes). We determined the set of differentially expressed genes based on the substrate used for growth of the Th strain: 566 genes were found to be upregulated during glucose fermentation, and 527 genes were upregulated in the presence of cellulose. These differentially expressed genes, including the genes related to carbohydrate depolymerisation classified as CAZyme genes, along with their relative expression levels and fold changes are shown in Supplementary Information 4 for all species classifications. Figure 5a shows the distribution of classified genes in the principal CAZy class (GH, AA, GT, CBM, and CE) according to the species and treatment, along with the precise distribution of CAZy genes classified as upregulated according to the treatment (Fig. 5b). The set of differentially expressed genes shows a variety of CAZymes that are species- and treatment-specific. The GH3 family, with β-glucosidase (EC 3.2.1.21) and xylan 1,4-β-xylosidase (EC 3.2.1.37) activity, was identified in all species under the glucose condition and only in Ta and Th under cellulose conditions. Two genes of the GH17 family (THAR02_07292 and THAR02_00771), with glucan endo-1,3-β-glucosidase (EC 3.2.1.39) and glucan 1,3-β-glucosidase (EC 3.2.1.58) activity, were differentially expressed in Th under the cellulose culture condition, and only one gene was expressed under the glucose condition in Tr (THAR02_10190). The GH3 family (β-glucosidase (EC 3.2.1.21) activity appears in all species and presents a high number of differentially expressed genes. The presence of the AA family in the Th cellulose culture (AA3 for the THAR02_09860 gene and AA8 for THAR02_10149) and in the Tr and Ta glucose cultures (AA1, AA7 and AA8 in THAR02_00247, THAR02_02524, and THAR02_10149, respectively, for Tr and AA1 in THAR02_06442 for Ta) shows the influence of different auxiliary activity enzymes. The peptide and protein sequences encoding the proteins that were detected in the extracts were identified via transcriptome analysis to the respective *T. harzianum* gene IDs, (Supplementary Material 3) for each species. A set of 80 of these genes was selected to illustrate their complete classification, according to their CAZy classification, genomic location and expression levels based on transcriptome analysis. Some genes from proteins identified in the Th strain were not expressed at significant levels compared with the other species, including the GH3 family protein encoded by the THAR02_00656 gene, which displayed β-glucosidase activity and low expression levels. In contrast, the THAR02_01480 gene, which encodes an uncharacterized FAD-dependent oxidoreductase protein that catalyses the oxidation of neutral and basic D-amino acids into their corresponding keto-acids, is expressed at high levels. THAR02_06250, which encodes a protein with α-L-arabinofuranosidase activity, was expressed only in the Th strain. Supplementary Material 3 shows the genomic locations of some important genes. In these 11 groups, two or more genes were observed to have the same genomic location. These genes appear to be located in close proximity across the genome, such as THAR02_02133, THAR02_02134 and THAR02_02147. These genes are located in the JOKZ01000041.1 scaffold and include an uncharacterized protein, copper-dependent lytic polysaccharide monooxygenase (LPMO) from the AA9 family and Endo-1,4-β-xylanase (EC 3.2.1.8) from the GH11 family. This genomic region is likely associated with biomass conversion with a high concentration of CAZy genes in the *T. harzianum* genome^23^.

### Co-regulatory networks

**Figure 6:**
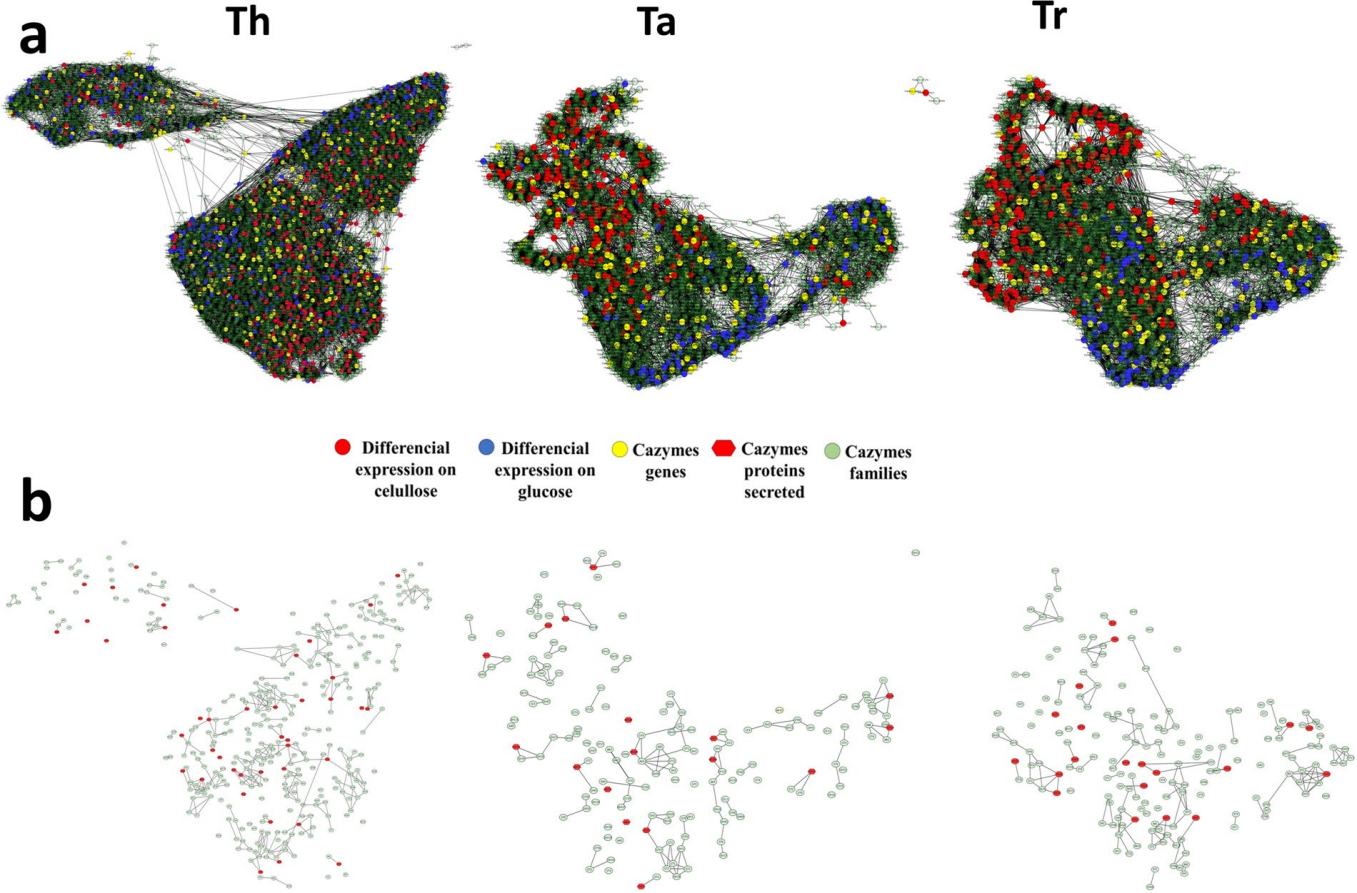
Co-regulation networks. (**a**) Complete co-regulation networks of Th, Tr and Ta. Red points indicate genes with differential expression on cellulose, Blue points indicate genes with differential expression on glucose, and Yellow points indicate CAZy genes. (**b**) Filtered co-regulation networks of Th, Tr and Ta, where all points are CAZy genes, and red points are genes encoding proteins secreted.

The co-regulatory RNA-Seq networks show the co-regulated CAZy genes and their relationships with the secreted and detected proteins. The graphs of the co-regulatory networks are shown in Fig. 6. The CAZy genes and the differentially expressed genes were distributed in the network (in Fig. 6a), and the co-regulation across CAZy genes is shown (in Fig. 6b, red points show the genes encoding proteins secreted: 41 Th genes, 20 Tr genes and 17 Ta genes). A total of 6521 nodes with 93,805 edges were obtained for the Th strain, 2591 nodes with 32,683 edges were obtained for the Ta strain, and 2842 nodes with 36,876 edges were obtained for the Tr strain. After selecting the CAZyme genes, the Th co-expression network showed 368 nodes and 310 edges, the Ta network displayed 166 nodes and 127 edges, and the Tr network included 183 nodes and 145 edges. The networks were partitioned into manageable clusters to explore the co-regulatory relationships. The cluster analysis classified 2565 genes in 30 clusters for Ta, 2840 genes in 26 clusters for Tr and 6516 genes in 57 clusters for Th. According to the CAZy genes classified in the cluster distribution, 367 genes in Th, 184 genes in Tr and 162 genes in Ta were observed. Supplementary Information 5 shows the full annotation of the cluster analysis (includes the replicon accession number, protein product, length, CAZy annotation, GH and CBM family, genome annotation, functional annotation, Pfam annotation, enzyme code, Gi number and secretome detection results). The cluster analysis more precisely investigated the contents of smaller groups of co-regulated genes and provided information that will be useful for understanding the relationships across fungal gene regulatory mechanisms.

## Discussion

In this study, we elucidated the genetic mechanisms of hydrolysis in three important species from the *Trichoderma* genus. We used several approaches to evaluate this complex biological system, including analyses of expression levels through RNA-Seq, secreted proteins under biomass degradation conditions, enzymatic activity and co-regulation networks based on gene expression. Our study provides unprecedented discoveries related to key enzymes that are common in different species of *Trichoderma* and, thus, fundamental in the process of vegetal biomass degradation. The variability observed in the *Trichoderma* enzymatic systems is mainly attributed to differences in substrate affinities or different lytic mechanisms. Each species presents a specific enzymatic activity profile that clearly corresponds to the substrate. Specific enzymes are regulated by cellulose as a substrate, generating higher levels of cellulose-dependent activities. In contrast, glucose enzyme activities were detected at lower levels due to the immediate consumption of glucose. According to the species classification, the enzymatic activity detected in aqueous extracts can be considered similar to those Tr and Th. The strain Ta showed a lower activity of cellulase but a higher activity of β-glucosidase (Fig. 2). The detected enzymatic activity is related to proteins secreted into the medium by the cells through different protein export or lysis mechanisms, and only the most stable proteins are detected in this environment at high levels. The similarity between these proteins enabled the sequence alignment of proteins from different *Trichoderma* spp. which revealed differences between proteins as minor as a single nucleotide that could explain the changes in the catalytic design of the binding site and enzymatic activity levels; thus, these small differences are very important for enzyme design. A bimodular GH7 protein, carbohydrate-binding module family 1 (CBM1), was detected in all species cultured in cellulose and only in the Tr strain cultured with glucose. According to the study by Momeni *et al*.^21^, the Cel7A enzyme (HjeCel7A), which is classified in the GH7 family, constitutes nearly half of the total protein secreted by *T. reesei*. Gene knock-out studies have shown that it is a rate-limiting factor in cellulose degradation, suggesting a key role for GH7 enzymes in biomass degradation by fungi^21^. Regarding the enzymatic activity, the species Ta had a different profile, and it is therefore interesting to note which proteins found only in this condition may respond to increased β-glucosidase activity (Supplementary Information 2). Forty-two proteins were identified, including β-glucosidases. Some appear to be very similar proteins, and A0A060DHV5_9HYPO has 94.6% identity with G9NS06_HYPAI and 91.8% with G9MUM1_HYPVG, with β-glucosidase (EC 3.2.1.21). It is observed that there are very similar proteins in databases: some differ by only one base pair and received different identification codes. This similarity should be noted, and only structural and functional tests can conclude the exact identity of a protein. However, in these cases, it was important to take these differences into account. The similarity between proteins from different studied species could be explained as a single nucleotide difference and could resize the catalytic design of the binding site, changing the enzymatic activity levels, making this small difference very important to enzymes design.

To detail the genes and genetic resources used by fungi, which made possible the secretion of the described proteins and their measured enzymatic activity, we discuss the total gene expression and the following exoproteome’s relationship with the transcriptome. As the transcriptional profile is very specific to cellular conditions, the use of cellulose and glucose as substrates allowed us to identify a distinct set of expressed genes, as described in Supplementary Information 4. Interestingly, the total number of genes shared in cultures grown in cellulose (Fig. 4b) is larger than that in cultures grown in glucose (Fig. 4c), indicating a specific group of genes related to cellulose metabolization in all the species. The number of identified genes was higher in Th than Ta or Tr because the reference genome from *T. harzianum* T6776, aligns best with Th and has more extensive genome coverage (78.22%). This analysis observed that higher levels of expression increase the probability of the protein having been detected at extraction, while lower levels of expression make the identification of the secreted protein more difficult. This same profile was described by Sun *et al*.^24^ who identified targets that were represented by highly expressed transcripts were more likely to be expressed as proteins (98–100% for the transcripts that show the highest expression levels) and concluded that approximately 10% of the transcripts that show low or no expression have their proteins expressed^24^.

The null values of expression observed for some genes, especially those related to species, could be explained by the catalytic function of these proteins, which may have been previously produced and remained in the extract after decreased or increased gene expression. In this way, the differences between techniques could show that the RNA-seq from 96 hrs of fermentation should not be exactly equal to the proteomic analysis, which could identify all the proteins accumulated in the aqueous extracts since the beginning of the fermentation. The data shown in Supplementary Information 3 are of great importance for the study of specific genes. The THAR02_00657 gene (αL-arabinofuranosidase activity) corresponds to a protein identified only in Tr, but the expression levels of this gene were significant for all the species and were higher for cultures with cellulose. The THAR02_00832 gene (Chitinase activity) shows significant expression levels for all species but is not classified as a differentially expressed gene, and the protein was identified only in the Tr extracts, with an expression level near that of the species Ta and 3 times lower than that described for Th under cellulose culture conditions. The identified genes related to the detected proteins were also analysed according to genome position to determine whether these proteins may have some type of co-regulatory relationship or very close physical locations in the total genomic sequence (Supplementary Material 3).These genomic regions were probably were associated with biomass conversion, a characteristic observed in previously work^23^. Different genes are positioned before, after and between the locations of these genes. These intermediates may be co-regulated with the observed genes, and thus, they are potentially important for cellulose and hemicellulose metabolism and degradation. These enriched genomic locations are used to improve point mutation techniques and to understand the influence of these genes on the effects of promoter region mutations or site-specific recombination strategies on enhancing cellulase synthesis and secretion.

To determine the genes that have a measurable relationship with each other, the exoproteoma and RNA-seq information was used to determine the co-expression networks. The networks showed the uninterrupted complex, synergistic relationships in the cells related to CAZymes (Fig. 6a) and the specific relationships between CAZymes (Fig. 6b). This first view of co-regulatory networks has never been reported for *T. harzianum*. The Ta and Tr co-expression networks were smaller than the Th network because these species exhibited a smaller number of classified genes compared with Th. In the cluster analysis, the contents of smaller groups of co-regulated genes were precisely evaluated, and information useful for understanding the relationships across fungal gene regulatory mechanisms was obtained.

## Conclusions

In conclusion, we use several biotechnological approaches to understand the different mechanisms of the hydrolysis of cellulose by *Trichoderma* spp., and we delimit a set of clusters of genes that must be co-regulated and be fundamental for this overall process of saccharification. The CAZymes analysis from co-regulatory networks revealed groups with a high degree of synergy as well as new genes involved in the production and secretion of the detected proteins and genes encoding hydrolytic enzymes. The data generated in this work can be used as a basis for more in-depth studies of the regulation between genes in co-regulatory networks. The elucidation of genetic relationships between the sets of genes provides important information for the development of recombinant microorganisms and simultaneously contributes to our understanding of the synergistic reactions among enzymes.

## Materials and Methods

### Cultivation

The species originated from the Brazilian Collection of Environment and Industry Microorganisms (CBMAI). The abbreviations Th, Ta and Tr are used to indicate the studied species throughout this manuscript. Th (IOC3844) was classified as *T. harzianum/lixii* and Ta (CBMAI0020) as *Trichoderma* spp., both of which display close phylogenetic proximity to *T. atroviride* CBS142.95. Tr (CBMAI 0711) was classified as *T. reesei* and corresponds to the wild-type species, for which other codes are employed in different collections (CCT 2768, ATCC 26921, QM 9414, and CBS 392.92).

The strains were grown on solid medium to produce a sufficient number of spores as an inoculum for fermentation. Th was grown on PDA (potato dextrose agar); Tr was grown on MEA medium (malt extract agar)^25^; and Ta was grown on MA2 (malt extract agar, 2% w/w)^26^. The plates were inoculated and grown for 8 days at 37 °C. After the plates were colonized, they were scraped with a washing solution to form the spore solution (500 µL/L Tween and 9 g/L NaCl). A Neubauer chamber was used to quantify the number of spores.

### Fermentation

Fermentation was performed in biological triplicates and was initiated with the inoculation of 10^7^ spores /mL in an initial volume of 200 mL of pre-inoculum composed of 10 g /L crystalline cellulose (Celuflok, São Paulo, Brazil, degree of crystallinity 0.72 g/g, composition 0.857 g/g cellulose and 0.146 g/g hemicellulose)^27^ or glucose, 1 g /L peptone, and 100 mL /L mineral base, pH 5.3 (buffer with potassium biphthalate). The composition of the mineral base was 20 g/L KH_2_PO_4_, 14 g/L NH_4_SO_4_ (1.4), 3 g/L MgSO_4_•7H_2_O, 3 g/L CaCl_2_•2H_2_O, 0.002 g/L CoCl_2_, 0.016 g/L MnSO_4_•H_2_O, 0.014 g/L ZnSO_4_•H_2_O, 0.05 g/L FeSO_4_•7H_2_O_4_ and 3 g/L urea. After 72 h of incubation, 50 mL of the pre-inoculum was used to inoculate 450 mL of fermentation solution in a 2 L Erlenmeyer flask. The composition of the fermentation volume varied according to the carbon source, which was either crystalline cellulose or glucose (10 g /L carbon source, 1 g /L peptone, 100 mL /L mineral base, 1 mL/L Tween, pH 5.3, with potassium biphthalate). The fermentation process continued for 96 h. The aqueous extract was frozen at −20 °C and the mycelium was quick-frozen in liquid nitrogen and stored at −80 °C.

### Enzyme activities

Cellulase activity was determined using the filter paper activity (FPA) test according to the method described by Ghose^28^. Xylanase activity was determined using the method described by Bailey and Poutanen^29^ measuring the release of reducing sugars as xylose for 10 min at 50 °C and pH 5.3 using the dinitrosalicylic acid. β-Glucosidase activity was measured with p-nitrophenol-b-D-glucoside (Sigma-Aldrich, St. Louis, USA) according to the method reported by Zhang *et al*.^30^. The assay was carried out using 20 µL of aqueous extracts and 80 µL of 12 mM pNPG, diluted in 50 mM citrate buffer (pH 4.8), and the mixture was incubated for 10 min at 50 °C. The reaction was stopped by adding 100 µL of 1 M Na_2_CO_3_, and the absorbance was measured at 400 nm. Protein levels were measured in microplates using a specific kit from Bio-Rad (Bio-Rad Laboratories, USA) using a procedure based on the Bradford method^31^.

### Phylogenetic analysis

Amplification of the ITS region (ITS1-5.8S-ITS2) from the genomic DNA of the species under study was conducted via PCR using ITS-1 and ITS-4 primers. The sequences were aligned using ClustalW^32^ implemented in the MEGA7 program^33^. The phylogenetic analyses were performed in MEGA7 using the neighbour-joining method^34^ with 1000 bootstrap replicates^35^ for each analysis. Pairwise deletion was employed to address alignment gaps and missing data.

The trees were visualized and edited using the Figtree program (http://tree.bio.ed.ac.uk/software/figtree/).

### RNA extraction

RNA was extracted from the Th and Ta mycelia samples using the LiCl RNA extraction protocol according to the method reported by Oliveira *et al*.^36^. The Tri Reagent Solution (Ambion) was used for the Tr samples according to the manufacturer’s instructions.

### High-throughput sequencing (RNA-Seq)

The libraries were constructed with RNA obtained from the mycelial samples using the Genome Analyser Illumina IIx according to the TruSEQ RNA sample preparation protocol v2^37^. The 18 biological triplicate samples were multiplexed for sequencing. After excluding the adapter and multiplex tag sequences, the RAW trimmed reads were 29 bp. The reads were deposited into the SRA database in NCBI under BioProject number PRJNA336221 and accession numbers SAMN06312791, SAMN06312792, SAMN06312793, SAMN06312794, SAMN06312795, and SAMN06312796 for Tr; SAMN06312797, SAMN06312798, SAMN06312799, SAMN06312800, SAMN06312801, and SAMN06312802 for Ta and SAMN06312803, SAMN06312804, SAMN06312805, SAMN06312806, SAMN06312807, and SAMN06312808 for Th.

CLCGenomicsWB 9.0 software was used to analyse the RNA-Seq data^38^. This software allowed the reads to be mapped to the *T. harzianum* T6776 genome^39^ using a length fraction of 0.5 and similarity of 0.8. The reads from different species were individually mapped to determine gene expression levels and the differentially expressed genes compared with the set of genes related to biodegradation reactions (CAZymes) provided in Supplementary Material 7, using a cutoff E-value of 10^−11^ ^40^. The expression data were log2 transformed and normalized with the following parameters to statistically analyse the differentially expressed genes: (1) fold change ≥ or ≤1.5 and (2) p-value ≤ 0.05. The reference genomes used in this analysis were *T. atroviride* JCM 9410 (BioProject PRJDB3574) *T. reesei* V2.0 JGI.

### Co-regulatory networks

The co-regulatory networks were assembled from the reference mapped RNA-Seq data using each set of biological triplicates. Genes showing null values for most of the replicates under different experimental conditions were excluded to diminish noise and to eliminate residuals in the analysis. A specific network for each species was assembled by calculating Pearson’s Correlation for each pair of genes. The highest reciprocal rank (HRR) method proposed by Mutwill *et al*.^41^ was used to empirically filter the edges, considering edges with an HRR less than or equal to 3. Thus, only edges representing the strongest correlations were selected. Genes classified as CAZy genes were selected for visual inspection. For the data analysis and network construction, Cytoscape software v 3.4.0^42^ was used. The cluster analysis procedure used the Heuristic Cluster Chiseling Algorithm (HCCA)^41^.

### RT-qPCR analysis

Reverse transcription-quantitative PCR (RT-qPCR) was performed, and the results are shown in Supplementary Information 8. Gene expression was quantified by continuously monitoring SYBR Green fluorescence. The reactions were performed in triplicate in a total volume of 6.25 µL. Each reaction contained 3.12 µL of SYBR Green Master Mix (Invitrogen, Carlsbad, CA), 1.0 µL of forward and reverse primers and 2.1 µL of diluted cDNA. The reactions were assembled in 384-well plates. PCR amplification-based expression profiling of the selected genes was performed using specific endogenous controls for each species, which are described in the Supplementary Material. RT-qPCR was conducted on an ABI PRISM 7500 HT (Applied Biosystems, Foster City, CA). Gene expression was calculated using the Delta-Delta cycle threshold method^43^. The obtained RT-qPCR results were compared with the RNA-Seq results from the assemblies generated (Supplementary Information 6). The fold changes of the selected genes exhibited the same expression profile in the RT-qPCR and RNA-Seq analyses.

### Exoproteome analysis using MSE

The analysis of secreted proteins was performed via liquid chromatography tandem mass spectrometry (LC-MS/MS) using the data-independent method of acquisition MS^E^. Triplicate samples from culture supernatants were pooled and purified by centrifugation followed by membrane filtration steps until a final concentration of 1–2 µg/µL was obtained. After centrifugation at 12,000 g and 4 °C for 15 min, the supernatant was recovered and concentrated using a 3-kDa Amicon Ultra™ filter (Millipore Merck, Darmstadt, HE, USA) and then washed three times with 50 mM ammonium bicarbonate buffer (pH 8.0). Proteins were quantified using a 2D Quant kit (GE Healthcare). Protein samples of 50 µg were processed for mass spectrometry (MS) according to a previously reported method (Murad, Souza, Garcia, & Rech, 2011). The proteins were treated with 0.2% (v/v) RapiGest SF (Waters Corp., Milford, MA, USA) and then reduced with 100 mM dithiothreitol (DTT) (GE Healthcare) and alkylated with 300 mM iodoacetamide (Sigma-Aldrich Co.). Sequencing-grade porcine trypsin (Promega Corp., Madison, WI, USA) was used for protein digestion at a 1:100 enzyme:protein ratio. The resulting tryptic peptides were recovered through 5% (v/v) trifluoroacetic acid (TFA) (Thermo Scientific) incubation, and the supernatants were transferred to glass vials. A solution of yeast alcohol dehydrogenase (ADH) (Waters − 186 002 328) was added to each vial at a final concentration of 125 µmol/µL and used as an internal calibrant for sample normalization.

Tryptic peptides analysis was performed using a nanoACQUITY UPLC system coupled to a Synapt G1 High-Definition Mass Spectrometer (Waters). A nanoflow ESI source with a lock spray source for lock mass measurements was employed during all chromatographic runs. Samples of approximately 3 µg of digested protein were desalted using a Trap Symmetry C18 column (Waters). The mixture of trapped peptides was then separated via elution using a water/ACN 0.1% (v/v) formic acid gradient through a Symmetry C18 capillary column (150 µm int. diam.). Data were acquired in the expression mass spectrometry mode (MSE), and multiple charged peptide ions (+2 and +3 and +4) were automatically mass selected and dissociated in the MS/MS experiments. The typical LC and ESI conditions included a flow of 1.8 µL per min, a nanoflow capillary voltage of 3 kV and a cone voltage of 30 V. Three analytical replicates per sample were analysed and processed individually.

The LC-MS/MS data were processed using ProteinLynx 3.0.1 v (Waters, UK), and the processed files (.pkl) were subjected to searches against the *Trichoderma* sequence database available in the UniProt Knowledgebase (UniProtKB; http://www.uniprot.org/uniprot/). Venn diagrams were employed to compare the identified protein profiles between samples using http://bioinformatics.psb.ugent.be/webtools/Venn/.

To make it possible to identify secreted proteins in the transcriptome, it was necessary to classify the proteins according to the names of the genes from which they are transcribed. For this purpose, the fasta sequences of the identified proteins were subjected to BLAST comparisons against the *T. harzianum* T6776 genome. A set of 80 proteins were detected in the extracts and had corresponding gene IDs.

## Electronic supplementary material

Supplementary Information 1

Supplementary Information 2

Supplementary Information 3

Supplementary Information 4

Supplementary Information 5

Supplementary Information 6

Supplementary Information 7

Supplementary Information 8
