# Author Correction: Network of proteins, enzymes and genes linked to biomass degradation shared by *Trichoderma* species

**DOI:** 10.1038/s41598-018-35990-4

**Published:** 2018-11-29

**Authors:** Maria Augusta Crivelente Horta, Jaire Alves Ferreira Filho, Natália Faraj Murad, Eidy de Oliveira Santos, Clelton Aparecido dos Santos, Juliano Sales Mendes, Marcelo Mendes Brandão, Sindelia Freitas Azzoni, Anete Pereira de Souza

**Affiliations:** 1 Center for Molecular Biology and Genetic Engineering (CBMEG), University of Campinas (UNICAMP), Campinas, SP Brazil; 2 University Unit of Biology, West Zone State University (UEZO), Rio de Janeiro, RJ Brazil; 3 Bioethanol Science and Technology Laboratory (CTBE), Brazilian Center of Research in Energy and Materials (CNPEM), Campinas, SP Brazil; 4 Department of Plant Biology, Biology Institute, University of Campinas (UNICAMP), Campinas, SP Brazil

Correction to: *Scientific Reports* 10.1038/s41598-018-19671-w, published online 22 January 2018

This Article contains an error in Figure 3b where the blue bars for ‘Th’ are incorrectly marked as ‘Tr’. The correct Figure 3 appears below as Figure 1.

**Figure 1:**
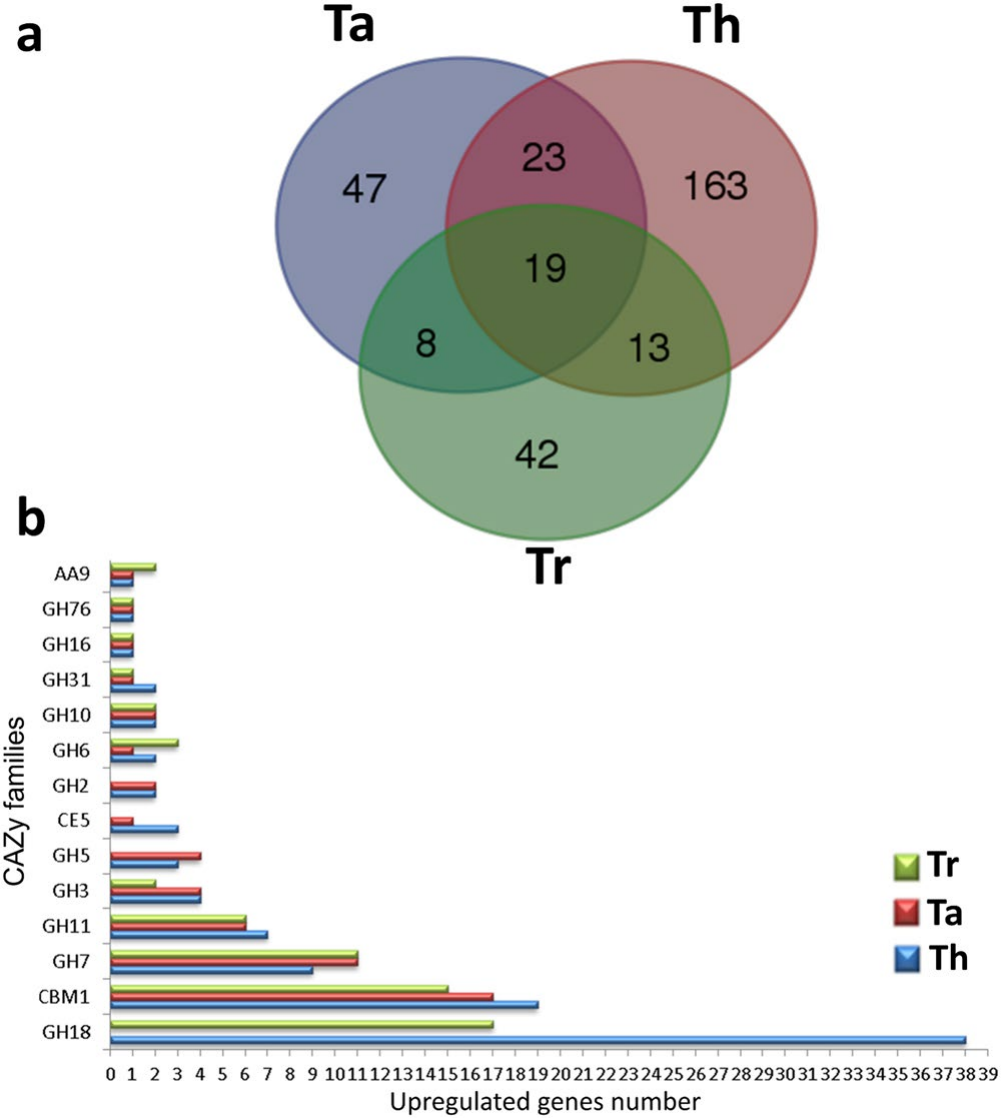
Protein classification by exoproteome analysis. (**a**) Venn diagram comparing proteins detected in the cultures of *Trichoderma* species. (**b**) Distribution of CAZymes among the secreted proteins of Tr, Ta, and Th under both fermentative conditions.

